# Management of severe pneumonia in respiratory non-intensive care unit: a retrospective study from a single center experience

**DOI:** 10.3389/fmed.2025.1666800

**Published:** 2025-09-18

**Authors:** Lucrezia Pisanu, Marianna Russo, Maria Arminio, Lorenzo Arlando, Valentina Conio, Francesco Rocco Bertuccio, Klodjana Mucaj, Mitela Tafa, Giulia Maria Stella, Angelo Guido Corsico

**Affiliations:** ^1^Cardiothoracic and Vascular Department, Unit of Respiratory Diseases, IRCCS Policlinico San Matteo Foundation, Pavia, Italy; ^2^Department of Internal Medicine and Medical Therapeutics, University of Pavia Medical School, Pavia, Italy

**Keywords:** pneumonia, biomarker, ventilation, personalized medicine, antibiotics

## Abstract

**Aim:**

Severe pneumonia management in the hospital setting often relies heavily on established clinical practice and physician experience. This approach has the purpose of enabling early identification of risk factors most strongly associated with severe pneumonia at the time of hospital admission.

**Methods:**

This retrospective study analyzed inpatients with pneumonia treated in a Respiratory disease unit, stratifying them into two groups—severe and non-severe pneumonia — according to the 2007 IDSA/ATS criteria, identifying differences in demographic profiles, clinical features, treatment strategies, and prognostic outcomes.

**Results:**

Out of a cohort of 302 patients, 26 (8.6%) met the criteria for severe pneumonia. A statistically significant difference was observed in the Pneumonia Severity Index (PSI > 90), recorded in 61.53% of patients with severe pneumonia compared to 41.31% in non-severe cases. The Charlson Comorbidity Index (CCI ≥ 4), indicative of lower 10-year survival due to comorbidities, was significantly more frequent in the severe group (84.61% vs. 61.23%). Microbiological analysis of bronchoalveolar lavage (BAL) showed a positivity rate of 75% in the severe group versus 35.48% in the non-severe group (*p* < 0.05). Significant differences were also found in the use of respiratory support: high-flow nasal cannula (HFNC) was used in 69.23% of severe cases versus 32.97% in non-severe cases, while CPAP was administered in 23.07% versus 5.43%, respectively. Finally, antibiotic therapy was significantly longer in the severe group, with a mean duration of 14.69 days compared to 11.77 days in non-severe patients, involving both intravenous and oral regimens as part of initial or sequential treatment.

**Conclusion:**

Timely recognition of these factors is essential to ensure optimal patient care, facilitate close monitoring of critically ill individuals, allow for prompt therapeutic escalation, and support ICU admission when needed. This analysis highlights the need for a critical reassessment of existing guidelines and underscores the value of integrating them with real-world clinical experience.

## Introduction

Pneumonia is a common infection of the lung parenchyma, and it represents even today a leading cause of hospital admission.

Clinical diagnosis is based on symptoms, physical examination, chest imaging and blood tests.

Exams involving etiology (urinary antigen testing, cultures from sputum or bronchoalveolar lavage, respiratory viral polymerase chain reaction, blood cultures) are important to optimize treatment if bacteria, virus or - less frequently - fungi and parasites are identified. However, a causal pathogen is often not detected (1).

The management of pneumonia in a hospital setting is very often based on common clinical practice and experience.

Clinical practice guideline for diagnosis and treatment of adults with community-acquired pneumonia (CAP) was approved by the American Thoracic Society (ATS) and the Infectious Diseases Society of America (IDSA) in 2019 (2, 3).

To define severe CAP (sCAP) it uses the ATS/IDSA CAP severity criteria from the 2007 statement (4).

In 2023 the European Respiratory Society (ERS), European Society of Intensive Care Medicine (ESICM), European Society of Clinical Microbiology and Infectious Diseases (ESCMID), and Latin American Thoracic Association (ALAT) launched a task force to produce the first international guidelines for sCAP.

In these recommendations, sCAP is accepted terminology used to describe patients with CAP that require admission to the intensive care unit (ICU), as they might need organ support.

One major or three minor criteria to follow, along with a combination of antibiotics for empirical treatment, were part of the former ATS/IDSA criteria for ICU admission.

However, as criterion for ICU admission can be heterogeneous in the absence of shock or need for mechanical ventilation, recommendations for this population should be cautiously provided (5–8).

## Materials and methods

This retrospective study included in patients with pneumonia that received care in the Respiratory disease unit of IRCCS Policlinico San Matteo Foundation in Pavia from October 2023 to October 2024.

This cohort comprised both CAP and healthcare-associated pneumonia (HCAP), that were analyzed together due to the small number of HCAP cases.

Data collection concerned demographics (gender, age, body mass index), home respiratory supports (oxygen, CPAP, NIV), home therapies (immunosuppressant, bronchodilators), history of dysphagia, respiratory and non-respiratory comorbidities, clinical information (type of pneumonia, severity scores, presence of pleural effusion) and hospital management (respiratory support, pharmacological treatment, procalcitonin measurement). Assessment and monitoring of pneumonia during hospitalization were based on biochemical markers and radiological imaging; pulmonary function tests were not performed during the acute phase but were recommended at discharge for patients with known or suspected respiratory comorbidities.

The analysis was conducted by selecting two groups of patients, those with severe pneumonia and patients with non-severe pneumonia.

The characterization of severe pneumonia was based on 2007 Infectious Diseases Society of America/American Thoracic Society Criteria for Defining Severe Community-acquired Pneumonia.

The study compared the two groups, with the purpose of recognize significative differences between demographics, clinical data, standard of treatment and prognostic values (duration of antibiotic therapy, length of hospital stay, in-hospital mortality).

The data were analyzed by the Z score for two population proportions (*^a^*) - a common statistical test used to determine if the difference between two proportions is statistically significant, indicating how many standard deviations the observed difference deviates from the expected difference under the assumption of no true effect - and the Mann-Whitney test (*^b^*) – a non-parametric test used to compare two independent groups, often employed as an alternative to the independent samples *t*-test when the assumption of normality is not met - in https://www.socscistatistics.com.

A *P*-value of <0.05 was considered statistically significant.

## Results

On a total of 302 patients, 26 patients met the criteria for severe pneumonia. 276 remaining patients were included in non-severe pneumonia group.

P/F was ≤ 250 in 24 patients; 17 patients present multilobar infiltrates; 8 patients presented confusion or disorientation at admission; blood test showed uremia ≥20 mg/dl in all 26 patients and leukopenia in 5 cases.

The other minor criteria for severe pneumonia were not found in the patient cohort.

Clinical and demographic data of patients are described in Table 1, comparing the severe pneumonia group and the non-severe pneumonia group.

**TABLE 1:** Clinical and demographic data in the severe pneumonia group and in the non-severe pneumonia group.

		Severe pneumonia	Non-severe pneumonia	*P*-value
	**Total patients**	26 (100%)	276 (100%)	
Type of pneumonia	CAP	23 (88.46%)	259 (93.84%)	0.28914 (^a^)
	HCAP	3 (11.54%)	17 (6.15%)	0.28914 (^a^)
Pneumonia severity scores	CURB-65 ≥ 2 (mortality risk > 6.8%, recommendation per derivation study: inpatient hospitalization)	10 (38.46%)	71 (25.72%)	0.16152 (^a^)
	PSI > 90 (moderate and high risk)	16 (61.53%)	114 (41.31%)	**0.0466 (^a^)**
	qSOFA ≥ 2 (high risk for in-hospital mortality)	1 (3.84%)	4 (1.44%)	0.35238 (^a^)
	Pleural effusion	10 (38.46%)	109 (39.49%)	0.92034 (^a^)
Gender	FEMALES	12 (46.15%)	131 (47.46%)	0.89656 (^a^)
	MALES	14 (53.85%)	145 (52.53%)	0.89656 (^a^)
	Mean age at hospital admission (age)	71.84	66.44	0.09492 (^b^)
BMI	<18.5	2 (7.69%)	16 (5.79%)	0.69654 (^a^)
	≥30	4 (15.38%)	38 (13.76%)	0.8181 (^a^)
	Dysphagia	2 (7.69%)	20 (7.24%)	0.93624 (^a^)
Respiratory support at home	Oxygen	5 (19.23%)	49 (17.75%)	0.8493 (^a^)
	CPAP	2 (7.69%)	9 (3.26%)	0.25014 (^a^)
	NIV	0 (0%)	6 (2.17%)	0.45326 (^a^)
Home therapy	Glucocorticoid	4 (15.38%)	30 (10.86%)	0.48392 (^a^)
	Other immunosuppressant	5 (19.23%)	21 (7.61%)	**0.04338 (^a^)**
	Chemotherapy	1 (3.84%)	6 (2.17%)	0.57548 (^a^)
	Bronchodilator therapy	10 (38.46%)	82 (29.71%)	0.35758 (^a^)
Respiratory comorbidities	COPD	9 (34.61%)	69 (25%)	0.28462 (^a^)
	Asthma	1 (3.84%)	17 (6.15%)	0.63122 (^a^)
	Bronchiectasis	2 (7.69%)	14 (5.07%)	0.56868 (^a^)
	ILD	0 (0%)	13 (4.71%)	0.25848 (^a^)
	Previous pneumonia	2 (7.69%)	65 (23.55%)	0.06148 (^a^)
	CCI ≥ 4 (estimated 10-year survival ≤ 53%)	22 (84.61%)	169 (61.23%)	**0.01778 (^a^)**

CAP, community-acquired pneumonia; HCAP, healthcare-associated pneumonia - patients who have multiple risks for being colonized by nosocomial multidrug-resistant pathogens (hospitalization for ≥ 2 days within the preceding 90 days, residence in a nursing home or extended care facility, home infusion therapy, chronic dialysis, home wound care and contact with subjects colonized by multidrug-resistant pathogens); CURB-65, CURB-65 Score for Pneumonia Severity estimates mortality of community-acquired pneumonia to help determine inpatient vs. outpatient treatment; PSI, Pneumonia Severity Index for CAP estimates mortality for adult patients with community-acquired pneumonia; qSOFA, Quick SOFA Score for Sepsis identifies high-risk patients for in-hospital mortality with suspected infection outside the ICU; BMI, body mass index; CPAP, continuous positive airway pressure; NIV, non-invasive ventilation; COPD, chronic obstructive pulmonary disease; ILD, interstitial lung disease; CCI, Charlson Comorbidity Index predicts 10-year survival in patients with multiple comorbidities.

### Pneumonia severity scores

Pneumonia severity scores were calculated retrospectively and were not routinely employed during the initial patient assessment. Notably, a significant difference between the two patient groups emerged only with the Pneumonia Severity Index (PSI), which was greater than 90 (moderate and high risk of mortality) in 61.53% of patients with severe pneumonia and in 41.31% of those with non-severe pneumonia.

### Demographics

There was no statistically significant difference in mean age, gender, and BMI between the two groups.

### Home therapy

Regarding home therapy, no significant differences were observed in respiratory treatment, while the only statistically significant difference concerned the use of immunosuppressive drugs (used chronically in 19.23% of patients with severe pneumonia).

### Comorbidities

Patients with severe pneumonia presented respiratory comorbidities in 11 cases (COPD, asthma, bronchiectasis, ILD), and 2 patients had previous pneumonia. Non-severe pneumonia patients had respiratory comorbidities in 103 cases, 65 patients had previous pneumonia. There were no statistically significant differences between the two groups.

**FIGURE 1:**
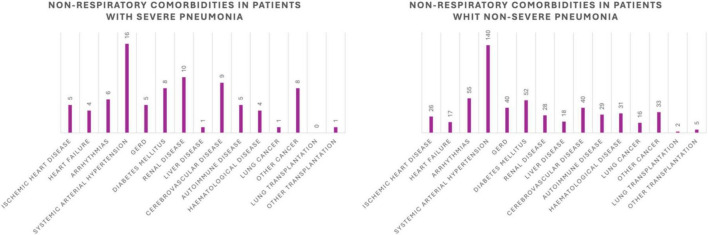
Distribution of non-respiratory comorbidities in the severe pneumonia group and in the non-severe pneumonia group (*n*).

Non-respiratory comorbidities are graphically represented in Figure 1 (ischemic heart disease, heart failure, arrhythmias, systemic arterial hypertension, gastroesophageal reflux disease, diabetes mellitus, renal disease, liver disease, cerebrovascular disease, autoimmune disease, hematological disease, lung cancer, other cancer, lung transplantation, other transplantation).

Charlson Comorbidity Index (CCI), that predicts 10-year survival in patients with multiple comorbidities, was greater than or equal to 4 in 22 patients of the severe pneumonia group (84.61%) and in 169 patients of the non-severe pneumonia group (61.23%). The comparison of this variable between the two groups was statistically significant.

### Etiology

Among the investigations performed to identify the etiology of pneumonia, bronchoalveolar lavage (BAL) was carried out in 8 patients with severe pneumonia and in 62 with non-severe pneumonia, with no statistically significant difference between the two groups. However, a statistically significant difference was observed in the microbiological results of bronchoalveolar lavage between the two groups, with a positivity rate of 75% in patients with severe pneumonia compared to 35.48% in those with non-severe forms (Table 2).

**TABLE 2:** Performance of bronchoalveolar lavage (BAL) and corresponding results.

	Severe pneumonia	Non-severe pneumonia	*P*-value
Total patients	26 (100%)	276 (100%)	
BAL performed	8 (30.76%)	62 (22.46%)	0.33706 (^a^)
BAL positive	75%	35.48%	**<0.00001 (^a^)**

The most frequently identified pathogens were *Candida*, virus, *Pseudomonas aeruginosa*, *Aspergillus*, *Haemophilus influenzae*, *Klebsiella pneumoniae*, *Escherichia coli*, *Staphylococcus aureus*, Enterobacter, *Stenotrophomonas*, *Serratia*, *Enterococcus faecalis*, *Corynebacterium*, *Mycobacterium intracellulare*.

### Respiratory support

In the severe pneumonia group, high-flow nasal cannula (HFNC) was utilized in 18 patients – 4 of which were already in home oxygen therapy.

6 patients required helmet continuous positive airway pressure (CPAP), 1 of which was already in home therapy with oxygen and CPAP for obstructive sleep apnea syndrome (OSAS). 4 patients necessitated non-invasive ventilation (NIV), 2 of which had home oxygen therapy.

The comparison between the two patient groups revealed a statistically significant difference in the use of HFNC (69.23% in severe pneumonia versus 32.97% in non-severe pneumonia) and CPAP (23.07% in severe pneumonia versus 5.43% in non-severe pneumonia), while no significant difference was observed in the utilization of NIV.

### Pharmacological treatment

In the severe pneumonia group, 47.36% of patients that needed bronchodilators and inhaled corticosteroids (on a total of 19 patients) were in bronchodilator therapy at home.

Systemic corticosteroids were provided in 22 patients, of which 13.63% of patients was in chronic treatment with glucocorticoids and 45.45% in inhalation therapy.

In the non-severe pneumonia group, 37.91% of patients that needed bronchodilators and inhaled corticosteroids (on a total of 211 patients) were in bronchodilator therapy at home.

Systemic corticosteroids were provided in 165 patients, of which 16.36% of patients was in chronic treatment with glucocorticoids and 38.18% in inhalation therapy.

No significant difference was found in this regard between the two groups.

The antibiotic therapy in severe pneumonia was administered for a mean duration of 14.69 days, using intravenous and oral antibiotics (as part of main therapy or as second treatment cycle), against a mean duration of 11.77 days in the non-severe pneumonia group, with this difference being statistically significant.

The antibiotics most used were cephalosporines, antipseudomonal penicillin, fluoroquinolones, tetracyclines, macrolides, amikacin, trimethoprim/sulfamethoxazole, clindamycin, beta-lactams, vancomycin, carbapenems, linezolid, metronidazole, aztreonam.

Table 3 summarizes the management differences between the two groups.

**TABLE 3:** Comparison of non-invasive respiratory supports, duration of antibiotic therapy, treatment with bronchodilators and corticosteroids, length of hospital stay and in-hospital mortality.

	Severe pneumonia	Non-severe pneumonia	*P*-value
Total patients	26 (100%)	276 (100%)	
High-flow nasal cannula (HFNC)	18 (69.23%)	91 (32.97%)	**0.00298 (^a^)**
Continuous positive airway pressure (CPAP)	6 (23.07%)	15 (5.43%)	**0.00072 (^a^)**
Non-invasive ventilation (NIV)	4 (15.38%)	18 (6.52%)	0.09894 (^a^)
Mean duration of antibiotic therapy (days)	14.69	11.77	**0.03752 (^b^)**
Bronchodilators and inhaled corticosteroids in bronchodilator therapy at home	47.36%	37.91%	0.17702 (^a^)
Systemic corticosteroids in chronic treatment with glucocorticoids	13.63%	16.36%	0.5892 (^a^)
Systemic corticosteroids in chronic inhalation therapy	45.45%	38.18%	0.29834 (^a^)
Length of hospital stay ≥ 10 days	22 (84.61%)	211 (76.44%)	0.34212 (^a^)
Mean duration of hospital stay (days)	18.15	15.10	0.06724 (^b^)
Death	2 (7.69%)	5 (1.81%)	0.05876 (^a^)

### Procalcitonin measurement

Procalcitonin levels may aid in distinguishing bacterial co-infections in patients with viral pneumonia or infections caused by intracellular pathogens. Moreover, monitoring procalcitonin levels during therapy, when considered alongside clinical assessment, may help reduce the duration of antibiotic treatment.

**FIGURE 2:**
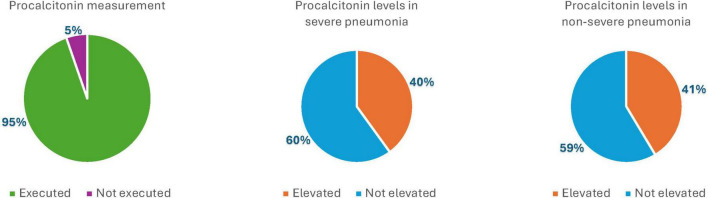
Graphic representation of procalcitonin measurement and elevation in the severe pneumonia group and in the non-severe pneumonia group (%).

Procalcitonin, which was routinely measured in most patients hospitalized for pneumonia (286 of 302 inpatients), was not generally used as a guiding parameter for initiating, adjusting, or modifying antibiotic therapy. It resulted elevated in 10 of 25 patients with severe pneumonia (40%) and in 108 of 261 patients with non-severe pneumonia (41.37%) – Figure 2 – and the difference between the two groups was not significative.

### Prognostic values

No significant differences were observed in terms of mean duration of hospital stay, with an average of 18.15 days for severe pneumonia and 15.10 days for non-severe cases.

Length of hospital stay was ≥10 days in 22 patients with severe pneumonia (13 patients were discharged at home, 8 patients have been transferred to rehabilitation and 10 patients to hospice, 2 patients died) and in 211 patients with non-severe pneumonia (224 patients were discharged at home, 36 patients have been transferred to rehabilitation, 6 patients to other non-ICU department and 5 patients to hospice, 5 patients died).

The mortality rate in severe cases was 7.69%, compared to 1.81% in non-severe cases, although this difference was not statistically significant.

## Discussion

Severe pneumonia is often managed in Respiratory non-intensive care unit, requiring constant monitoring of the patient’s condition.

Knowledge of respiratory system pathophysiology, non-invasive respiratory support systems, and pharmacological treatment management enables pulmonologists to successfully treat severe forms of pneumonia.

To this end, it is important to recognize the risk factors most strongly associated with severe pneumonia as early as the patient’s arrival. This is essential to ensure that patients receive the highest standard of care and that the most critically ill patients are promptly identified and closely monitored. This allows for timely therapeutic escalation and facilitates prompt ICU admission when necessary.

### Etiology

The findings related to the use of BAL highlight its potential importance in the management of severe pneumonia, as it facilitates the administration of targeted antibiotic therapy, thereby allowing for a more precise treatment strategy and greater therapeutic success.

### Respiratory support

When looking at the 2023 ERS/ESICM/ESCMID/ALAT guidelines - although these guidelines were not the primary framework guiding the study, they provided the foundation for assessing essential aspects of pneumonia management - the data from this study align well with them in terms of the use of HFNC, which is a highly effective oxygen support that ensures accurate delivery of the set FiO_2_, generates a flow-dependent upper airway washout effect enhancing CO2 clearance in the anatomical dead space, and enhances tolerance through gas conditioning and a comfortable interface (5, 9).

### Pharmacological treatment

The analysis of steroid use results more complex, as it is heavily influenced by the individual physician’s experience and often shaped by biases related to indications other than shock - a condition, however, not present in this cohort of patients.

### Procalcitonin measurement

The results of this study indicate that use of procalcitonin as a guiding biomarker remains largely dependent on the physician’s clinical judgment. As such, its role in antibiotic management has almost always been considered alongside other parameters and integrated into a broader assessment of the overall clinical picture (10–16).

## Conclusion

This study contributes to enhancing awareness of pneumonia management in respiratory wards and fosters a deeper understanding and more effective application of clinical guidelines in daily practice.

Although the cohort of 302 patients is not very large – the study was conducted retrospectively and does not follow a case-control design - several significant findings were obtained in the statistical analysis of the results.

The analysis encourages a critical appraisal of existing guidelines and advocates for their integration with the practical insights gained through everyday clinical experience.

In the event of a future concrete definition of pneumonia monitoring parameters and early identification of risk factors, artificial intelligence could cross-analyze all the data and generate results with significant prognostic value.

## Data Availability

The original contributions presented in this study are included in this article/supplementary material, further inquiries can be directed to the corresponding author. The data that support the findings of this study are available on request from the corresponding author.

## References

[1] Grief S Loza J. Guidelines for the evaluation and treatment of pneumonia. *Prim Care.* (2018) 45:485–503. 10.1016/j.pop.2018.04.001 30115336 PMC7112285

[2] Metlay J Waterer G Long A Anzueto A Brozek J Crothers K Diagnosis and treatment of adults with community-acquired pneumonia. An official clinical practice guideline of the American thoracic society and infectious diseases society of America. *Am J Respir Crit Care Med.* (2019) 200:e45–67. 10.1164/rccm.201908-1581ST 31573350 PMC6812437

[3] Armstrong C. Community-acquired pneumonia: updated recommendations from the ATS and IDSA. *Am Fam Physician.* (2020) 102:121–4. 32667166

[4] Mandell L Wunderink R Anzueto A Bartlett J Campbell G Dean N Infectious diseases society of America/American thoracic society consensus guidelines on the management of community-acquired pneumonia in adults. *Clin Infect Dis.* (2007) 44:S27–72. 10.1086/511159 17278083 PMC7107997

[5] Martin-Loeches I Torres A Nagavci B Aliberti S Antonelli M Bassetti M ERS/ESICM/ESCMID/ALAT guidelines for the management of severe community-acquired pneumonia. *Eur Respir J.* (2023) 61:2200735. 10.1183/13993003.00735-2022 37012080

[6] Ramirez J Wiemken T Peyrani P Arnold F Kelley R Mattingly W Adults hospitalized with pneumonia in the United States: incidence, epidemiology, and mortality. *Clin Infect Dis.* (2017) 65:1806–12. 10.1093/cid/cix647 29020164

[7] Rögnvaldsson K Bjarnason A Ólafsdóttir I Helgason K Guðmundsson A Gottfreðsson M. Adults with symptoms of pneumonia: a prospective comparison of patients with and without infiltrates on chest radiography. *Clin Microbiol Infect.* (2023) 29:108.e1–e6. 10.1016/j.cmi.2022.07.013 35872174

[8] Zilberberg M Nathanson B Puzniak L Zilberberg N Shorr A. Descriptive epidemiology of hospitalized patients with bacterial nosocomial pneumonia who experience 30-day readmission in the US, 2014-2019. *PLoS One.* (2022) 17:e0276192. 10.1371/journal.pone.0276192 36490261 PMC9733878

[9] Maggiore S Jaber S Grieco D Mancebo J Zakynthinos S Demoule A High-flow versus venturimask oxygen therapy to prevent reintubation in hypoxemic patients after extubation: a multicenter randomized clinical trial. *Am J Respir Crit Care Med.* (2022) 206:1452–62. 10.1164/rccm.202201-0065OC 35849787

[10] Niederman M. Biological markers to determine eligibility in trials for community-acquired pneumonia: a focus on procalcitonin. *Clin Infect Dis.* (2008) 47:S127–32. 10.1086/591393 18986278

[11] Summah H Qu J. Biomarkers: a definite plus in pneumonia. *Mediators Inflamm.* (2009) 2009:675753. 10.1155/2009/675753 20011658 PMC2786247

[12] Bessat C Boillat-Blanco N Albrich W. The potential clinical value of pairing procalcitonin and lung ultrasonography to guide antibiotic therapy in patients with community-acquired pneumonia: a narrative review. *Expert Rev Respir Med.* (2023) 17:919–27. 10.1080/17476348.2023.2254232 37766614

[13] Ebrahimi F Giaglis S Hahn S Blum C Baumgartner C Kutz A Markers of neutrophil extracellular traps predict adverse outcome in community-acquired pneumonia: secondary analysis of a randomised controlled trial. *Eur Respir J.* (2018) 51:1701389. 10.1183/13993003.01389-2017 29519921

[14] Maves R Enwezor C. Uses of procalcitonin as a biomarker in critical care medicine. *Infect Dis Clin North Am.* (2022) 36:897–909. 10.1016/j.idc.2022.07.004 36328642

[15] Cheng C Chien M Su S Yang S. New markers in pneumonia. *Clin Chim Acta.* (2013) 419:19–25. 10.1016/j.cca.2013.01.011 23384502 PMC7094281

[16] Israelsen S Fally M Tarp B Kolte L Ravn P Benfield T. Short-course antibiotic therapy for hospitalized patients with early clinical response in community-acquired pneumonia: a multicentre cohort study. *Clin Microbiol Infect.* (2023) 29:54–60. 10.1016/j.cmi.2022.08.004 35988851

